# Transcriptomics of a KDELR1 knockout cell line reveals modulated cell adhesion properties

**DOI:** 10.1038/s41598-019-47027-5

**Published:** 2019-07-23

**Authors:** Andrea Blum, Saleem Khalifa, Karl Nordström, Martin Simon, Marcel H. Schulz, Manfred J. Schmitt

**Affiliations:** 1 Molecular and Cell Biology, Department of Biosciences (FR 8.3) and Center of Human and Molecular Biology (ZHMB), Saarland University, D-66123 Saarbrücken, Germany; 2 Cluster of Excellence, Multimodal Computing and Interaction, Saarland University and Department for Computational Biology and Applied Algorithmics, Max Planck Institute for Informatics, Saarland Informatics Campus, Saarbrücken, Germany; 3 Genetics/Epigenetics, Center for Human and Molecular Biology, Saarland University, Saarbrücken, Germany; 4 Molecular Cell Biology and Microbiology, Wuppertal University, D-42097 Wuppertal, Germany; 5 Institute for Cardiovascular Regeneration, Goethe University Frankfurt, 60590 Frankfurt am Main, Frankfurt, Germany

**Keywords:** Extracellular matrix, Protein transport

## Abstract

KDEL receptors (KDELRs) represent transmembrane proteins of the secretory pathway which regulate the retention of soluble ER-residents as well as retrograde and anterograde vesicle trafficking. In addition, KDELRs are involved in the regulation of cellular stress response and ECM degradation. For a deeper insight into KDELR1 specific functions, we characterised a KDELR1-KO cell line (HAP1) through whole transcriptome analysis by comparing KDELR1-KO cells with its respective HAP1 wild-type. Our data indicate more than 300 significantly and differentially expressed genes whose gene products are mainly involved in developmental processes such as cell adhesion and ECM composition, pointing out to severe cellular disorders due to a loss of KDELR1. Impaired adhesion capacity of KDELR1-KO cells was further demonstrated through *in vitro* adhesion assays, while collagen- and/or laminin-coating nearly doubled the adhesion property of KDELR1-KO cells compared to wild-type, confirming a transcriptional adaptation to improve or restore the cellular adhesion capability. Perturbations within the secretory pathway were verified by an increased secretion of ER-resident PDI and decreased cell viability under ER stress conditions, suggesting KDELR1-KO cells to be severely impaired in maintaining cellular homeostasis.

## Introduction

The secretory pathway represents a highly organised multi-organelle compartment which enables the transport of newly synthesised secretory proteins as well as membrane proteins to their final cellular destination and place of activity. These transport processes include massive membrane fluxes from one compartment to another and need to be counterbalanced by tightly connected cellular control mechanisms. A central key player in the regulation of vesicle transport from and to the Golgi is the eukaryotic KDEL receptor (KDELR), a seven transmembrane protein which was initially described to be responsible for the retrieval of soluble ER residents from the Golgi back to the ER^1–3^. This process is largely driven by pH differences between two compartments: the slightly acid pH environment of the Golgi allows binding of KDEL-bearing chaperones, subsequently initiating KDELR interaction with Arf-GAP and coatomer components to trigger COPI-mediated retrograde transport^4,5^. In contrast, the neutral pH in the ER lumen causes the ligand/receptor-complex to dissociate and KDELRs are recycled back to the Golgi via anterograde transport^3,6^. Initially described in yeast, three KDELR homologues (KDELR1, KDELR2, and KDELR3) with different ligand specificities and expression profiles were subsequently identified in mammalian cells^2,7,8^. Further studies revealed an important signalling function of mammalian KDEL receptors in order to balance membrane fluxes and to maintain Golgi homeostasis. Thereby, KDELR activation by ligand binding causes an interaction with Golgi-associated G proteins: Gq mediates the activation of Src family kinases (SFKs) and, thereby, stimulates anterograde transport processes to the plasma membrane^9^. This Golgi-to-plasma membrane trafficking was recently shown to be likewise facilitated by an interaction of KDELR with the monomeric Gα_0_ subunit, subsequently activating Rab1/Rab3 GTPases^10^. An interaction with Gs results in PKA activation and promotion of retrograde vesicle trafficking. For longer-term regulation of both transport directions, PKA further activates transcription factors to enhance the expression of genes encoding COPI and COPII components^11^.

Due to their relevance in controlling intracellular transport processes, KDELRs are associated with cell stress conditions and the regulation of a cellular stress response, while its particular outcome highly depends on the KDELR expression level and the respective cell type^12–14^. In this context, a specific mutation in KDELR1 affecting its interaction with protein phosphatase 1 (PP1) leads to an increased stress response and enhanced apoptosis of T-cells^15^. In contrast, KDELR overexpression has been demonstrated to result in MEK1/ERK-dependent autophagy activation, enabling the cell to minimise the accumulation of proteins such as SOD1, α-synuclein, and huntingtin which are associated with neurodegenerative diseases^13^. In addition, KDELRs have also been shown to regulate invapodia formation and extracellular matrix degradation in a Src family kinase-mediated signal transduction process by activating focal adhesion kinases (FAK)^16,17^.

Furthermore, recent studies also suggest a regulated transport pathway of KDELRs to the plasma membrane which might include novel signalling functions at the cell surface after extracellular ligand binding^18–20^. To facilitate the analysis of such novel KDELR functions, we focused on a genome-wide characterisation of a KDELR1-knockout (KO) HAP1 cell line. Transcriptome analysis revealed strong alterations in the expression of genes whose products are involved in cellular development, adhesion and extracellular matrix (ECM) composition. In this regard, differences concerning the adhesion and migration behaviour of KDELR1-KO cells could be confirmed by *in vivo* scratch and adhesion assays. Furthermore, enhanced secretion of protein disulphide isomerase (PDI) and increased sensitivity to ER stress were also observed as characteristic KDELR1-KO phenotype.

## Material and Methods

### Cultivation of cells

HAP1 represents a near-haploid human cell line derived from the male chronic myelogenous leukemia (CML) cell line KBM-7 containing a single copy of each chromosome, except for a heterozygous 30-megabase fragment of chromosome 15^21^. KDELR1-KO was generated via the CRISPR/Cas technology using ATGAATCTCTTCCGATTCCT as guide RNA sequence. KDELR1-KO cells maintain a 1 bp deletion in exon 1 which causes a frameshift and thereby a non-functional KDELR1 gene. Both, HAP1 wild-type and HAP1 KDELR1-KO cells were commercially obtained from the company Horizon which provides additional information about the cell line and the experimental approach to generate a particular KO strain on the following homepage: https://www.horizondiscovery.com.

HAP1 cells were cultivated in IMDM (Iscove’s Modifed Dulbecco’s Medium) medium (Gibco) supplemented with 10% fetal bovine serum (FCS, Biochrom) and 1% penicillin/streptomycin (Pen/Strep, Sigma) at 37 °C and 5% CO_2_.

### Sequencing of genomic KDELR1 DNA

10 µl cell suspension was transferred into a reaction tube, washed once with 1 × PBS and resuspended in sterile water before freezing at −80 °C overnight. 1 µl of the cell lysate was used as PCR template to amplify the genomic KDELR1 sequence within its first exon. The reaction was carried with the „Phusion Hot Start II HighFidelity DNA Polymerase“-Kit (Thermo Scientific) and the following primers: forward: AGCTCCAGCCTTTGCTCCCCCTCCCAAA, reverse: CCCAAACCCTTCCTGAGTCC TGCGACGT. The PCR product was cleaned up using the „Wizard® SV Gel and PCR CleanUp System“ (Promega) and sequenced by “GATC Biotech AG”.

### RNAseq

RNA isolation was performed with the Direct-zol RNA MiniPrep Kit (Zymo Research) which includes DNAse digestion. RNA integrity was checked by denaturing gel electrophoresis. We enriched for poly-A RNA using „NEBNext^®^ Poly(A) mRNA Magnetic Isolation Modules“ and created strand specific cDNA libraries using the „NEBNext^®^ Ultra^TM^ II Directional RNA Library Prep Kits for Illumina^®^“ (New England BioLabs) using 1 µg total input RNA and 8 PCR cycles. Quality of the cDNA libraries was determined via Bioanalyzer (Agilent Bioanalyzer 2100). Sequencing of the samples was carried out in a „HiSeq. 2500 Platform” (Illumina) using high-output mode (100nt SE reads). Reads were demultiplexed with bcl2fastq (v1.8.4) and trimmed for adaptor contamination and low-quality bases with the cutadapt (v1.4.1) wrapper trim_galore (v0.3.3)^22^.

### Bioinformatics analysis of RNAseq data

Salmon software^23^ was used for the quantification of gene expression levels in HAP1 wild-type and KDELR1-KO cells. We used gene annotation from the Ensembl database^24^ version 95 for the analysis with Salmon. Using estimated gene expression counts from Salmon, DESeq. 2 software was used to estimate differentially expressed genes^25^. p-values have been corrected for multiple testing using the Benjamini-Hochberg procedure.

For the analysis of Gene Ontology (GO) enrichment we used the Ontologizer software (version 2.1)^26^ using the GO annotation database version 1.2^27^.

### Scratch assay

1 × 10^6^ HAP1 wild-type and KDELR1-KO cells were seeded in 6-well plates containing IMDM medium and incubated at 37 °C and 5% CO_2_. After reaching approximately 80% confluency, the cell network was slightly disintegrated by a scratch in the middle of the well using a 1,000 µl pipette tip. The medium was changed to FCS-free IMDM, and the scratch area was measured immediately (=time point 0 h). After 24 h and 48 h incubation in FCS-free medium, scratch areas were measured and compared to the respective area at time point 0 which was set to 100%.

### Collagen and laminin coating of 96 well plates

Collagen type I (Sigma) was diluted in sterile water to a working concentration of 100 µg/ml. For the coating of 96-well plates, 100 µl of the working solution was added to each well and incubated at 37 °C for 5 h. After removal of the remaining solution, the coated surface was allowed to dry. Wells were washed with sterile water and dried for storage at room temperature until use. Laminin (Sigma) was diluted in 1 × PBS to a final concentration of 100 µg/ml, and 100 µl were added to each well of a 96-well plate. Plates were sealed to prevent evaporation and incubated at 37 °C for 5 h. The residing solution was removed, the wells were washed with 1 × PBS and covered with 1 × PBS for storage at 4 °C until use.

### Adhesion assay

2 × 10^4^ cells of HAP1 wild-type and its KDELR1-KO variant were seeded in collagen-coated, laminin-coated or uncoated wells of a 96-well plate with IMDM medium, respectively. After adhesion for 4 h, unbound cells were removed by washing with 1 × PBS. Adherent cells were fixed with 2% paraformaldehyde (PFA, in 1 × PBS) for 15 min, washed twice with 1 × PBS and stained with 0.1% crystal violet solution (Merck) in 10% ethanol for 10 min. After 5 washing steps with sterile water, absorption was measured in a plate reader „SpectraMax® Paradigm® Multi-Mode Microplate Platform“ (Molecular Devices) with the „Multi-Mode Analysis Software 2010“ (Molecular Devices).

### Cell viability assay

HAP1 wild-type and KDELR1-KO cells (5 × 10^4^ cells each) were seeded in 96-well plates with phenol red-free DMEM medium (Gibco) supplemented with 10% FCS and 1% Pen/Strep and cultivated for 24 h. Subsequently, cells were treated with 1 or 1.5 µg/ml thapsigargin (Sigma Aldrich, in DMSO) or with the corresponding amount of DMSO in the negative control sample. After 24 h incubation, cell viability was determined via MTT assay. In brief, a solution of MTT (3-(4,5-Dimethyl-2-thiazolyl)-2,5-diphenyl-2H-tetrazolium bromide, Sigma) was added to a final concentration of 0.4 g/l (in 1 × PBS) and the 96-well plates were incubated for 3 h at 37 °C and 5% CO_2_. Arosen crystals were dissolved by adding 100 µl solubilisation solution (Isopropanol 89.6%, HCl 0.4%, Triton X-100 10%) and slow shaking at 20 °C. Absorption was measured at 570 nm using „SpectraMax® Paradigm® Multi-Mode Microplate Platform“ (Molecular Devices) with the „Multi-Mode Analysis Software 2010“ (Molecular Devices). Calculation of mean values and standard deviations were performed with Microsoft Excel.

### Protein extraction and western analysis

HAP1 cells were incubated in IMDM medium (Gibco) without FCS for 24 h at 37 °C and 5% CO_2_. Supernatant was collected, dialysed against 1 × PBS for 48 h (ZelluTrans T2, Roth) and concentrated by centrifugation using VivaSpin columns (5 kDa MWCO, Sartorius). The total protein amount of the concentrated supernatant was determined by NanoDrop measurement (NanoDrop 2000c, PEQLAB) and equally adjusted for both samples. SDS-PAGE was performed in 10% Tris-Tricine gels under non-reducing conditions, and subsequent semi-dry blotting onto PVDF membranes was carried out in transfer buffer (25 mM Tris, 190 mM glycine, 0.1% SDS, 20% methanol). After transfer, membranes were blocked in 3% BSA in TBS-T (100 mM Tris-HCl, pH 7.5, 100 mM NaCl, 0.05% Tween20) overnight at 4 °C, and subsequently incubated with antibodies against PDI (Abcam (ab2792, [RL90]), 1:1,000 in 3% BSA/TBS-T) for 1 h at 20 °C. Incubation with HRP-coupled secondary antibody (anti-mouse-HRP, 1:10,000 in 3% BSA/TBS-T, Sigma) was performed for 1 h at 20 °C. Chemiluminescence reaction was performed by a short incubation with “SuperSignal^TM^ West Femto Maximum Sensitivity Substrate“ (Thermo Scientific) and signals were detected and quantified with an „Amersham Imager 600“ (GE Healthcare).

## Results and Discussion

### KDELR1-KO affects transcriptome of HAP1 cells

**Figure 1:**
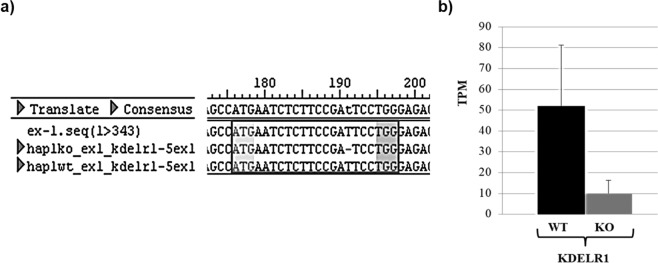
Confirmation of KDELR1-KO in HAP1 cells. (**a**) Sequencing result for the genomic KDELR1 DNA sequence of KDELR1-KO cells (third lane) and wild-type (fourth lane). KDELR1-KO cells show a deletion of „T16“ within the first exon of the KDELR1 gene in HAP1 cells, leading to a premature stop-codon in exon 2 (T188 G189 A190). (**b**) mRNA level of KDELR1 in wild-type (WT) and KDELR1-KO (KO) HAP1 cells. Represented are the mean values of the TPM (transcripts per million) from poly-A enriched RNA seq (triplicates with standard deviation).

KDELRs are multifunctional proteins with well-known functions in the retention of soluble ER-resident proteins as well as in the regulation of transport processes within the secretory pathway^1,2,9,11^. In addition, KDELRs were shown to contribute to the regulation of cellular stress response and ECM degradation^13,14,16^. Since our recent studies strongly indicated an additional co-localisation of KDELRs at the plasma membrane, a broader spectrum of novel KDELR functions can be predicted. To investigate such KDELR1-specific functions, we characterised a KDELR1-KO HAP1 cell line, which holds a deletion of thymine at position 16 within the genomic KDELR1 sequence that leads to a premature stop in translation, resulting in a KDELR1-KO (Fig. 1). Specificity of the gene knock-out in this KDELR1-KO cell line was further addressed by searching for undesired CRISPR/Cas9 off-targets in KDELR2 and KDELR3 through comparative RNAseq analysis between HAP1 wild-type and KDELR1-KO cells. By screening the corresponding bam-files at the nucleotide level, no mismatching could be detected in either RNA, except for some minor SNPs that were apparent both in wild-type cells and in the CRISPR generated KDELR1 knock-out cell line (data not shown). Therefore, any undesired off-targeting in the KDELR1-KO cell line seems rather unlikely, although we did not perform a genome-wide analysis to fully exclude such possibility.

**Figure 2:**
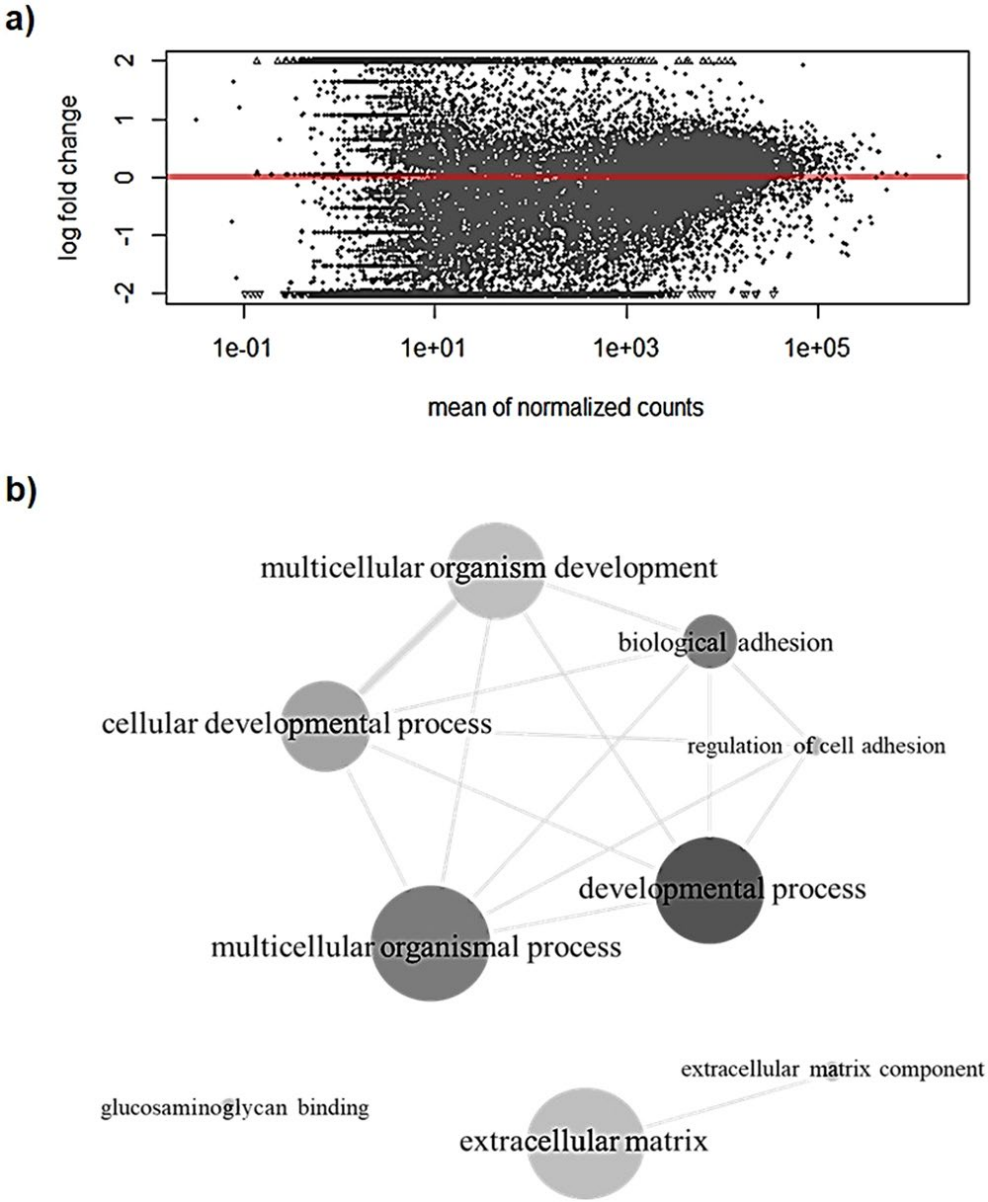
KDELR1-KO affects the transcriptome of HAP1 cells. (**a**) MA plot representing the difference between wild-type and KDELR1-KO samples. (**b**) Visualisation of significantly enriched GO terms (adjusted p-value < 0.01) in KDELR1-KO cells compared to HAP1 wild-type. GO terms and their adjusted p-values were used in REViGO to generate three interactive graphs (allowed similarity: medium (0.7), database: *Homo sapiens*, semantic similarity measure: SimRel), which are shown in one image. The size of the circles represents the frequency of the respective GO term in the gene set. More intensive grey shades were used to show higher significance in the GO analysis. Similar GO terms were connected with lines, whereby the width of each line demonstrates the level of similarity^32^.

For a more general overview of the possible consequences of a gene knockout in KDELR1, we performed a whole transcriptome analysis and compared HAP1 wild-type with KDELR1-KO cells. The analysis revealed over 300 differentially regulated genes (DEGs, adjusted p-value < 0.05, Supplementary Table S1) and, thus, demonstrated a strong effect on the transcriptome due to a loss of KDELR1. The MA plot (Fig. 2a) shows that KDELR1-KO samples clearly deviate from wild-type, presented by the high number of genes with larger fold changes. The respective mean of normalised counts additionally supposes considerable cellular effects caused by some of these changes. To obtain more detailed information on the changes in distinct biological processes, GO enrichment was performed which in total identified nine significantly enriched terms using DEGs with adjusted p-values < 0.01 (Fig. 2b). These DEGs are mainly involved in processes such as cell development, adhesion and extracellular matrix components and point to strong differences concerning the extracellular conditions. As the number of up- or down-regulated DEGs is nearly balanced within each term, we are not able to further specify the impact of the KDELR1-KO (Supplementary Table S2). Since it has already been shown that KDELRs activate focal adhesion kinases (FAK) and contribute to the regulation of ECM degradation, we hereby confirm the general importance of KDELR1 in such processes^16,17^.

### KDELR1-KO affects cellular migration and adhesion behaviour

**Figure 3:**
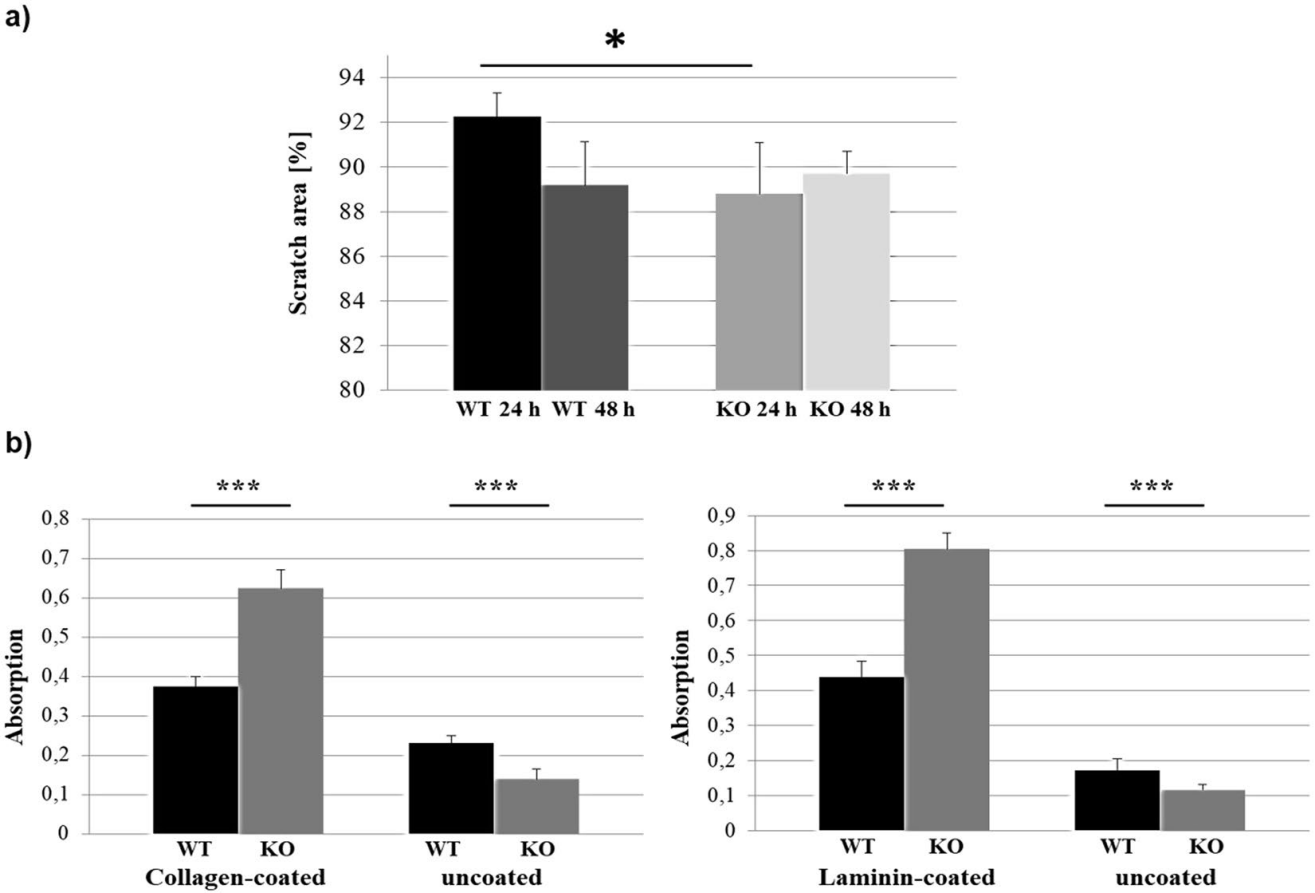
KDELR1-KO cells show enhanced migration/proliferation ability but impaired adhesion properties on uncoated surfaces which can be rescued by coating with collagen or laminin. (**a**) Scratch assay to compare migration behaviour of KDELR1-KO cells to HAP1 wild-type. Areas at time point 0 h were measured and calculated at 100%, scratch areas after 24 h and 48 h incubation in FCS-free medium were measured and presented in a diagram as mean value from 6 replicates. Error bars indicate the corresponding standard deviation, statistical significance was determined by students t-test and significant differences were marked by an asterisks (p < 0.05). Additionally, two-way ANOVA was performed which identified significant differences for samples (p = 0.047) and interaction (p = 0.009). (**b**) Adhesion assay to compare adhesion behaviour of KDELR1-KO to HAP1 wild-type cells in collagen-coated, laminin-coated or uncoated 96-well plates. Unbound cells were removed by washing with 1 × PBS. After 4 h adhesion time, adherent cells were fixed with 2% PFA, washed twice with 1 × PBS and stained with crystal violet. Absorption was measured at 590 nm after 5 washing steps with sterile water. Diagrams represent the mean value from 15 replicates and the respective standard deviation. Statistical significance was determined by students t-test and significant differences were marked by three asterisks (p < 0.01). Two-way ANOVA additionally indicated significant differences for the samples (p < 0.01), condition (p < 0.01), and interaction (p < 0.01).

As the GO enrichment revealed several biological processes that were significantly de-regulated in KDELR1-KO cells, the direct influence on the physiological phenotype of the cells was investigated. Therefore, we analysed the migration behaviour of KDELR1-KO cells based on scratch assays in comparison to HAP1 wild-type cells. For this purpose, the respective cells were seeded in 6-well plates and cultivated for 24 h allowing adhesion. Confluency of approximately 80% was aspired, and the cell network was subsequently disintegrated by a scratch. Further cell proliferation was avoided by continuing the cultivation in FCS-free culture medium, the scratch area was directly measured at time point 0 h and again after 24 h and after 48 h. Despite the usage of FCS-free medium, cell proliferation could not be completely avoided and, thus, the distinct differentiation between multiplying and propagating cells was hampered which must be considered when interpreting the results. Exemplary images of HAP1 wild-type and KDELR1-KO cells at time point 0 h and after 24 h are illustrated in Supplementary Fig. S3. After 24 h, KDELR1-KO cells showed a stronger migration/proliferation behaviour compared to wild-type cells, indicated by a significantly increased reduction of the scratch area. Only after 48 h, a comparable decrease of the scratch area could be determined for wild-type cells. Interestingly, for KDELR1-KO cells, we observed no further reduction of the scratch area after 48 h, but rather a slight increase, suggesting a starting cell detachment (Fig. 3a). However, it should be noted that the earlier cell detachment observed at previous time points (after 24 h) - or in general a poorer cell adhesion capacity - could also promote cell migration of KDELR1-KO cells. To further investigate this phenomenon, we analysed the adhesion behaviour and seeded cells in uncoated, collagen-coated or laminin-coated 96-well plates. After 4 h, wells were washed to remove unbound cells and crystal violet staining was performed to quantify the amount of adherent cells. Thereby it could be demonstrated that in case of uncoated surfaces, the adhesion properties of KDELR1-KO cells were clearly impaired compared to wild-type (Fig. 3b).

As the process of cell adhesion is mediated by ECM components, changes in ECM composition itself might have an influence on cellular adhesion ability. In KDELR1-KO cells, significant changes in ECM-related processes could be detected at the transcriptional level. Since KDELRs are essential for the regulation of vesicle transport, KDELR1-KO presumably affects the secretion of ECM components. It is already known that especially the secretion of large fibrillar procollagen requires a precise functionality of COPII-mediated transport and that any disturbance of COPII components leads to defects in ECM assembly^28–30^.

To rescue the assumed defects in ECM composition, surface coating with both collagen and laminin was performed which not only improved but nearly doubled the adhesion capacity of KDELR1-KO in comparison to wild-type cells (Fig. 3b). To compensate for impaired binding conditions, KDELR1-KO cells might upregulate other cellular players of adhesion processes to ensure at least basal cell binding ability. Indeed, the transcriptome analysis provides some significantly upregulated genes which could represent such candidates, for example cell adhesion molecule 1, contactin 3, platelet glycoprotein 4, homeobox protein Mohawk, protocadherin 17, protocadherin β-4, protocadherin β-5, prostaglandin G/H synthase 2, tyrosin protein kinase SYK, T-lymphoma invasion and metastasis-inducing protein 1, and brorin. We hypothesise that this permanent attempt to improve cell adhesion by ECM-defective KDELR1-KO cells could then result in an increased adhesion capability of KDELR1-KO cells on coated surfaces. The observed upregulation of platelet glycoprotein 4, a ligand of collagen, could for example explain the observed stronger adhesion of KDELR1-KO cells on collagen-coated surfaces. However, as we did not investigate defects in ECM composition biochemically, and KDELR1-KO cells additionally showed a large number of differentially expressed genes in general developmental processes, a more complex explanation for the observed phenotype might also be possible.

### KDELR1-KO cells show an increased secretion of PDI and enhanced sensitivity under ER stress conditions

**Figure 4:**
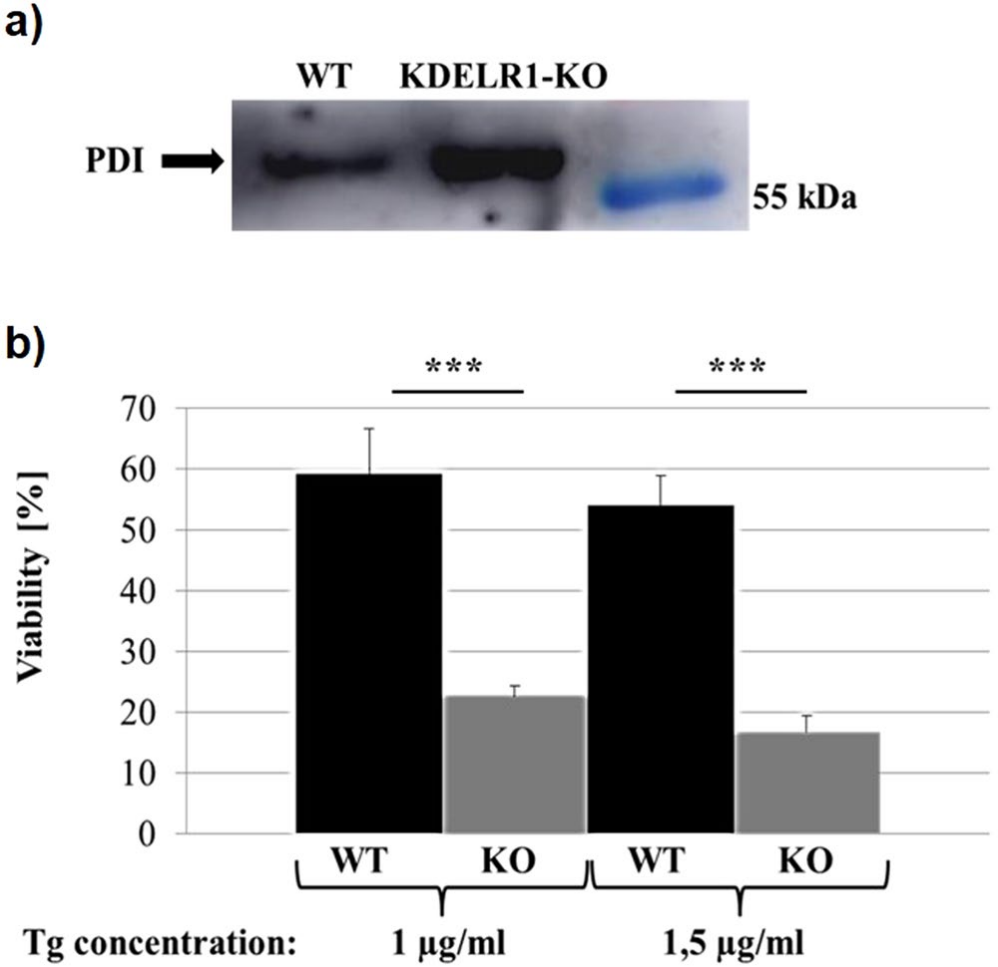
KDELR1-KO cells show an increased secretion of PDI and reduced cell viability under ER stress conditions. (**a**) Secretion of PDI in HAP1 wild-type (WT) and KDELR1-KO cells. In each case, a cell-free culture supernatant was dialysed against PBS, concentrated by VivaSpin centrifugation, and the total protein amount was determined and equally adjusted. For both samples, the same amount of protein was separated by SDS-PAGE and PDI signal intensity was subsequently analysed by western blotting. (**b**) Sensitivity of HAP1 WT and KDELR1-KO cells under ER stress conditions. Cells were treated with thapsigargin (Tg) for 24 h to induce ER stress and cell viability was analysed via MTT assays. Viability in DMSO control samples was calculated as 100%, viability of Tg-treated cells was determined and is represented in the diagram as mean value from 20 (1 µg/ml Tg) or 10 (1,5 µg/ml Tg) replicates together with the respective standard deviation. Statistical significance was determined by students t-test and significant differences were marked by three asterisks (p < 0.01). Additionally, two-way ANOVA was performed which indicated significant differences for the samples (p < 0.01), condition (p < 0.01), and interaction (p < 0.01).

Because of the important KDELR-functions in the regulation of vesicle trafficking within the secretory pathway, we further characterized KDELR1-KO HAP1 cells for defects concerning the ER-retention system and investigated secretion of the ER-resident KDELR ligand protein disulphide isomerase (PDI). We cultivated KDELR1-KO and wild-type cells in FCS-free medium for 24 h and collected the cell-free culture supernatant for subsequent dialysis and concentration. The amount of PDI detected by western analysis was nearly doubled in the supernatant of KDELR1-KO cells compared to wild-type (Fig. 4a). Since our transcriptome data did not show any significant upregulation of PDI isoforms at the mRNA level, it can be assumed that the detected protein increase of PDI in the supernatant of (untreated) KDELR1-KO cells results from enhanced ER chaperone secretion due to the loss of KDELR1 rather than being caused by a general increase of cellular stress response in this cell line. The retention of soluble ER-resident proteins represents the first and best described KDELR function^1,6,31^. Loss of KDELR was already demonstrated to result in an increased secretion of chaperones in yeast^1^. By expressing a KDELR1 variant with defects in ligand binding in HeLa cells, increased secretion of the ER-resident chaperone BiP could only be detected under ER-stress but not under regular non-stress conditions. The action of the endogenous wild-type KDELR1, which was not completely repressed by overexpression of the mutant, could probably be the reason for this observed minor effect^14^. In our studies, we could detect a stronger secretion of the KDELR ligand PDI in KDELR1-KO cells, confirming defects in directed protein transport even under non-stress conditions.

Since KDELRs are known to be involved in the regulation of cellular stress response and transport defects are also associated with cell stress, we assumed KDELR1-KO cells to have difficulties in counterbalancing stress conditions. To induce ER-stress, KDELR1-KO and HAP1 wild-type cells were incubated with the SERCA inhibitor thapsigargin for 24 h and subsequently analysed for cell viability using MTT assays. Indeed, a strong decrease in the viability of KDELR1-KO cells compared to wild-type was observed, indicating severe defects in the regulation of a cellular stress response (Fig. 4b). A typical cellular reaction to counterbalance ER-stress conditions is the upregulation of chaperones to increase the protein folding capacity within the ER. In case of a KDELR1 knockout, upregulated chaperones were not efficiently retained in the ER as demonstrated for PDI, indicating that the folding capacity might not be sufficient to adapt the increasing demand. However, it has already been described that KDELR1 malfunctions diminish the activation of the cell stress associated MAP kinases p38 and JNK1^14^, and that KDELR1 regulates eIF2α de-phosphorylation via PP1 interaction^15^. Regulation of cellular stress response in KDELR1-KO cells is, therefore, negatively affected at different levels, leading to substantial difficulties of a cell to compensate ER-stress conditions.

## Conclusions

Our study provides the first complete transcriptomic characterisation of a KDELR1-KO cell line to get a global insight into the defects of these cells. Within the analysed HAP1 cell line, a KDELR1-KO causes significant transcriptomic changes in four rather general developmental processes supporting the wide-ranging functions of KDELR1 in maintaining cellular homeostasis. Significant enrichment in five more specific GO terms concerning cell adhesion and ECM formation points to a vital necessity to counterbalance KDELR1 loss in these processes. We validated these findings by subsequent molecular analysis of protein secretion and cell adhesion. An impaired adhesion capacity of KDELR1-KO cells was confirmed by adhesion assays and could be rescued by collagen- or laminin-coating of the surface. The strongly increased adhesion ability of KDELR1-KO cells on coated surfaces compared to wild-type supports the transcriptomic changes to improve adhesion processes and compensate for corresponding defects. Since KDELRs are key players in regulating protein/vesicle trafficking processes within the secretory pathway, this transport might also be disturbed in KDELR1-KO cells. Here, mislocalisation of proteins of the secretory system was demonstrated by increased PDI secretion which might contribute to the observed increased sensitivity of KDELR1-KO cells to ER stress conditions. Thus, KDELR1-KO cells seem to be severely affected in cellular homeostasis and require compensation in cell adhesion processes at the transcriptional level. Considering the diverse expression level of KDELR1 and its various distinct functions in different cell types, other characteristics of a KDELR1-KO are likewise possible, especially for varying tissue types. Such analysis could represent an interesting and fruitful approach for future studies.

## Supplementary information

Supplementary Information

## Data Availability

All data analysed during this study are included in this published article (and its Supplementary Information Files). The raw dataset generated during transcriptome analysis is available in the European Nucleotide Archive (ENA) repository (accession number: PRJEB33498).
