# Supplementary material for: Transcriptomics of a KDELR1 knockout cell line reveals modulated cell adhesion properties
Source: Sci Rep. 2019 Jul 23;9:10611. doi: 10.1038/s41598-019-47027-5 (PMC6650600; doi:10.1038/s41598-019-47027-5)
Supplement: Supplementary file 1 — Supplementary Information [file 41598_2019_47027_MOESM1_ESM.pdf]

Transcriptomics of a KDELR1 knockout cell line reveals modulated cell adhesion properties

*Andrea Blum [a], Saleem Khalifa [b], Karl Nordström [c], Martin Simon [d], Marcel H. Schulz [b,e], *Manfred J. Schmitt [a]

[a]Molecular and Cell Biology, Department of Biosciences (FR 8.3) and Center of Human and Molecular Biology (ZHMB), Saarland University, D-66123 Saarbrücken, Germany.

[b]Cluster of Excellence, Multimodal Computing and Interaction, Saarland University and Department for Computational Biology and Applied Algorithmics, Max Planck Institute for Informatics, Saarland Informatics Campus

[c]Genetics/Epigenetics, Center for Human and Molecular Biology, Saarland University

[d]Molecular Cell Biology and Microbiology, Wuppertal University, D-42097 Wuppertal, Germany

[e]Institute for Cardiovascular Regeneration, Goethe University Frankfurt, 60590 Frankfurt am Main

Supplementary Table S1: DEGs of the transcriptome analysis comparing HAP1 wild-type and HAP1 KDELR1-KO cells

Gene-ID	Gene name	adjusted p-value	Fold-Change
ENSG00000180353.10	HCLS1	2,32E-70	-8,20919219
ENSG00000026025.15	VIM	2,24E-59	-5,0347944
ENSG00000186340.14	THBS2	9,49E-56	-6,444271646
ENSG00000145242.13	EPHA5	2,33E-51	7,158160571
ENSG00000185222.8	TCEAL9	1,11E-43	-9,157984695
ENSG00000178522.14	AMBN	9,50E-41	-4,125701454
ENSG00000197565.15	COL4A6	1,31E-40	-4,692394641
ENSG00000130303.12	BST2	5,90E-37	-8,238463683
ENSG00000052850.5	ALX4	1,49E-32	-5,301034698
ENSG00000152217.16	SETBP1	5,04E-29	-4,986919273
ENSG00000132718.8	SYT11	7,23E-29	-2,856189592
ENSG00000156299.13	TIAM1	3,09E-27	3,314667192
ENSG00000134802.17	SLC43A3	2,79E-26	-3,255057521
ENSG00000169594.13	BNC1	8,57E-26	3,337302104
ENSG00000092345.13	DAZL	9,43E-26	-4,048958829
ENSG00000136960.12	ENPP2	1,72E-23	-4,265987206
ENSG00000157064.10	NMNAT2	4,62E-23	4,099707417
ENSG00000224039.1	CDYLP1	7,25E-23	-4,443883553
ENSG00000284109.1	CDYLP1	7,25E-23	-4,443883553
ENSG00000221818.8	EBF2	1,13E-22	4,379950994
ENSG00000154645.13	CHODL	3,87E-21	3,524182313
ENSG00000172461.10	FUT9	2,71E-20	-6,161620907
ENSG00000050030.13	NEXMIF	1,16E-19	-4,429603346
ENSG00000134531.9	EMP1	1,58E-18	-3,796898784
ENSG00000147027.3	TMEM47	1,93E-17	3,54139651
ENSG00000136542.8	GALNT5	2,32E-17	3,15562015
ENSG00000150051.13	MKX	2,96E-17	3,666343008
ENSG00000104313.17	EYA1	8,07E-17	2,641365369
ENSG00000136859.9	ANGPTL2	6,34E-16	-4,755047396
ENSG00000101144.12	BMP7	8,79E-16	-9,184378464
ENSG00000137558.7	PI15	8,79E-16	-4,493403278
ENSG00000204262.11	COL5A2	8,79E-16	2,777971672
ENSG00000118246.13	FASTKD2	1,05E-15	2,638411958
ENSG00000117477.12	CCDC181	1,12E-15	3,593367315
ENSG00000133687.15	TMTC1	1,13E-15	3,09982207
ENSG00000180964.16	TCEAL8	8,95E-15	-9,655678938
ENSG00000153714.5	LURAP1L	1,25E-14	3,409262296
ENSG00000140563.14	MCTP2	8,16E-14	2,926461579
ENSG00000128918.14	ALDH1A2	1,53E-13	2,954190991
ENSG00000113805.8	CNTN3	3,78E-13	5,455882107
ENSG00000178403.3	NEUROG2	4,98E-13	2,193681171
ENSG00000165025.14	SYK	5,05E-13	2,306040027
ENSG00000124019.9	FAM124B	1,52E-12	-2,987057179
ENSG00000165323.15	FAT3	1,52E-12	-4,453288811

ENSG00000188730.4	VWC2	1,52E-12	3,816708215
ENSG00000282908.1	FAT3	1,52E-12	-4,453288811
ENSG00000186103.3	ARGFX	2,57E-11	-5,974439501
ENSG00000170775.2	GPR37	3,24E-11	1,708551779
ENSG00000213694.3	S1PR3	9,01E-11	2,622634285
ENSG00000080986.12	NDC80	1,81E-10	-2,054586556
ENSG00000155961.4	RAB39B	2,22E-10	-5,370799311
ENSG00000007062.11	PROM1	2,76E-10	2,44985619
ENSG00000173157.16	ADAMTS20	3,20E-10	3,529672966
ENSG00000155093.17	PTPRN2	3,71E-10	-9,343494164
ENSG00000060718.20	COL11A1	3,85E-10	-5,856246494
ENSG00000156475.18	PPP2R2B	6,62E-10	-1,846021285
ENSG00000135373.12	EHF	7,10E-10	-2,819513437
ENSG00000182968.4	SOX1	1,23E-09	2,934854404
ENSG00000138771.14	SHROOM3	1,28E-09	-2,046658826
ENSG00000038427.15	VCAN	1,33E-09	-2,771822208
ENSG00000004468.12	CD38	1,84E-09	2,958988024
ENSG00000117600.12	PLPPR4	1,87E-09	2,801732618
ENSG00000164651.16	SP8	1,93E-09	2,11792535
ENSG00000026508.17	CD44	2,13E-09	-3,635882504
ENSG00000183421.11	RIPK4	2,56E-09	3,379697219
ENSG00000156689.6	GLYATL2	4,16E-09	-2,941401181
ENSG00000234444.9	ZNF736	4,49E-09	-2,522187761
ENSG00000188227.12	ZNF793	6,41E-09	-1,879122299
ENSG00000163536.12	SERPINI1	7,65E-09	2,510119009
ENSG00000183117.18	CSMD1	7,75E-09	-1,861917181
ENSG00000212747.4	RTL8B	8,12E-09	-2,335614413
ENSG00000164120.13	HPGD	9,24E-09	1,869581141
ENSG00000198398.2	TMEM207	1,39E-08	3,297616809
ENSG00000144410.4	CPO	1,53E-08	2,77396218
ENSG00000067445.20	TRO	2,75E-08	-3,386895505
ENSG00000151789.10	ZNF385D	2,79E-08	-5,162765145
ENSG00000183807.7	FAM162B	2,79E-08	2,128875282
ENSG00000038295.7	TLL1	3,16E-08	2,782313082
ENSG00000138944.7	KIAA1644	3,16E-08	-2,246579395
ENSG00000120675.5	DNAJC15	3,56E-08	-3,360531258
ENSG00000154040.20	CABYR	3,82E-08	1,890197566
ENSG00000114315.3	HES1	3,83E-08	-1,632061252
ENSG00000170370.11	EMX2	5,70E-08	2,999212322
ENSG00000198354.6	DCAF12L2	6,80E-08	-7,114818738
ENSG00000105438.8	KDELR1	7,49E-08	-2,307905645
ENSG00000164112.12	TMEM155	9,90E-08	-8,372205904
ENSG00000135917.13	SLC19A3	1,22E-07	1,828919506
ENSG00000196781.14	TLE1	1,43E-07	1,737540117
ENSG00000127507.17	ADGRE2	2,09E-07	-2,313982897
ENSG00000189056.13	RELN	2,20E-07	-2,260017977
ENSG00000101605.12	MYOM1	2,78E-07	1,834984623

ENSG00000131018.22	SYNE1	3,50E-07	1,629489533
ENSG00000105894.11	PTN	4,34E-07	7,993379508
ENSG00000166265.11	CYYR1	4,34E-07	-1,487847657
ENSG00000146215.13	CRIP3	4,57E-07	2,721786442
ENSG00000138119.16	MYOF	4,79E-07	-2,689944938
ENSG00000282230.1	ADAM9	4,84E-07	-9,771329227
ENSG00000175538.10	KCNE3	4,96E-07	-2,517244009
ENSG00000114200.9	BCHE	6,39E-07	2,142711186
ENSG00000180806.4	HOXC9	7,00E-07	-2,546087755
ENSG00000172554.11	SNTG2	7,83E-07	1,364715751
ENSG00000171617.13	ENC1	8,86E-07	-2,363231662
ENSG00000261787.1	TCF24	9,56E-07	2,079687529
ENSG00000164107.8	HAND2	1,04E-06	1,791465735
ENSG00000187957.7	DNER	1,04E-06	2,463684386
ENSG00000180660.7	MAB21L1	1,08E-06	1,685520694
ENSG00000047648.21	ARHGAP6	1,35E-06	1,980810894
ENSG00000164695.4	CHMP4C	1,36E-06	-3,203613096
ENSG00000134343.12	ANO3	1,47E-06	2,933573051
ENSG00000134330.18	IAH1	2,34E-06	1,654124812
ENSG00000146938.14	NLGN4X	2,34E-06	-7,720372999
ENSG00000107562.16	CXCL12	2,58E-06	7,627208709
ENSG00000197608.11	ZNF841	3,02E-06	-2,198238479
ENSG00000154589.6	LY96	3,08E-06	2,825159757
ENSG00000016402.13	IL20RA	3,13E-06	1,638919206
ENSG00000174473.15	GALNTL6	3,16E-06	1,962486634
ENSG00000125266.6	EFNB2	3,64E-06	-2,163770325
ENSG00000165973.18	NELL1	3,71E-06	-2,032162183
ENSG00000133106.14	EPSTI1	3,72E-06	-4,69593448
ENSG00000120658.13	ENOX1	3,78E-06	-1,828624177
ENSG00000138792.9	ENPEP	3,89E-06	-2,793741186
ENSG00000070915.9	SLC12A3	4,17E-06	-4,606574847
ENSG00000123612.15	ACVR1C	4,54E-06	3,150502464
ENSG00000173930.8	SLCO4C1	5,28E-06	2,172181735
ENSG00000112562.18	SMOC2	6,12E-06	-3,755424553
ENSG00000177570.13	SAMD12	7,56E-06	2,130404127
ENSG00000269190.5	FBXO17	8,04E-06	2,483010586
ENSG00000282954.1	FBXO17	8,04E-06	2,483010586
ENSG00000164796.17	CSMD3	8,29E-06	1,8192867
ENSG00000196109.6	ZNF676	9,24E-06	-7,405771704
ENSG00000142871.16	CYR61	9,31E-06	-1,51961135
ENSG00000155530.2	LRGUK	9,37E-06	1,774508801
ENSG00000135547.8	HEY2	9,60E-06	5,476322571
ENSG00000178573.6	MAF	1,02E-05	1,290124143
ENSG00000135218.17	CD36	1,06E-05	1,907951223
ENSG00000113209.8	PCDHB5	1,15E-05	1,480839899
ENSG00000154330.12	PGM5	1,19E-05	1,720891193
ENSG00000178053.17	MLF1	1,43E-05	-3,824184867

ENSG00000134954.14	ETS1	1,47E-05	1,434203949
ENSG00000241166.6	HLA-L	1,76E-05	-21,12903774
ENSG00000156076.9	WIF1	1,87E-05	-7,250531303
ENSG00000197584.11	KCNMB2	2,29E-05	-1,790632628
ENSG00000128714.5	HOXD13	2,35E-05	1,559741577
ENSG00000170162.13	VGLL2	2,57E-05	6,053639772
ENSG00000260027.4	HOXB7	3,01E-05	-6,757189325
ENSG00000105143.12	SLC1A6	3,05E-05	-2,202066817
ENSG00000114455.13	HHLA2	3,93E-05	-2,08237609
ENSG00000163347.5	CLDN1	4,20E-05	1,30705518
ENSG00000111335.12	OAS2	4,22E-05	-2,038032145
ENSG00000120833.13	SOCS2	4,42E-05	-1,840720452
ENSG00000133019.11	CHRM3	5,15E-05	2,11184731
ENSG00000100234.11	TIMP3	5,25E-05	-1,482401651
ENSG00000144229.11	THSD7B	5,63E-05	-2,063834695
ENSG00000046889.18	PREX2	5,76E-05	-2,058898634
ENSG00000197705.9	KLHL14	6,30E-05	1,837302613
ENSG00000118971.7	CCND2	6,49E-05	-1,542459098
ENSG00000180730.4	SHISA2	6,78E-05	1,361627769
ENSG00000188385.11	JAKMIP3	6,98E-05	-2,667364021
ENSG00000163631.16	ALB	7,31E-05	2,393744492
ENSG00000146197.8	SCUBE3	7,36E-05	-1,197722706
ENSG00000179344.16	HLA-DQB1	7,52E-05	4,352078733
ENSG00000219438.8	FAM19A5	7,55E-05	-7,123259612
ENSG00000082497.11	SERTAD4	7,91E-05	1,607344647
ENSG00000134769.21	DTNA	7,91E-05	-2,486491283
ENSG00000281230.2	SERTAD4	7,91E-05	1,607344647
ENSG00000059804.15	SLC2A3	8,00E-05	1,370062986
ENSG00000134853.11	PDGFRA	8,72E-05	-1,962848852
ENSG00000144681.10	STAC	9,25E-05	1,377070936
ENSG00000078018.19	MAP2	9,41E-05	2,092569786
ENSG00000186854.10	TRABD2A	0,000101958	-1,30290113
ENSG00000198131.13	ZNF544	0,000101958	2,413000548
ENSG00000118946.11	PCDH17	0,000105366	1,376030134
ENSG00000075388.3	FGF4	0,000117648	-2,27627795
ENSG00000104237.7	RP1	0,000122604	2,17962717
ENSG00000144339.11	TMEFF2	0,000122604	2,085213107
ENSG00000147145.12	LPAR4	0,000122604	2,007748999
ENSG00000107242.17	PIP5K1B	0,000125572	2,081098838
ENSG00000134202.10	GSTM3	0,000131295	1,588939623
ENSG00000121742.16	GJB6	0,000133662	1,320666818
ENSG00000112164.5	GLP1R	0,000153857	-3,016657311
ENSG00000272398.5	CD24	0,000162101	1,635424251
ENSG00000135046.13	ANXA1	0,000180439	1,656850355
ENSG00000152583.12	SPARCL1	0,000189992	-2,059114019
ENSG00000225972.1	MTND1P23	0,000195058	-2,155266015
ENSG00000152779.13	SLC16A12	0,000198382	2,85745643

ENSG00000146374.13	RSPO3	0,000204707	-3,848348902
ENSG00000146072.6	TNFRSF21	0,000206807	-1,117483425
ENSG00000104611.11	SH2D4A	0,000209733	1,616945446
ENSG00000164344.15	KLKB1	0,000209733	2,40903816
ENSG00000164093.15	PITX2	0,000212382	1,950326266
ENSG00000110848.8	CD69	0,00024532	2,307304856
ENSG00000235268.2	KDM4E	0,000246036	-6,767690837
ENSG00000113532.12	ST8SIA4	0,000250891	1,923426951
ENSG00000073756.11	PTGS2	0,000252896	1,86846534
ENSG00000000005.5	TNMD	0,000302621	-1,609197925
ENSG00000204959.4	ARHGEF34P	0,000307839	2,746715861
ENSG00000179083.6	FAM133A	0,00030965	2,11810259
ENSG00000145824.12	CXCL14	0,000310692	-1,634414033
ENSG00000007372.21	PAX6	0,000326011	-1,53273734
ENSG00000179008.8	C14orf39	0,000333856	1,714494084
ENSG00000163064.6	EN1	0,000373191	-2,296233999
ENSG00000138135.6	CH25H	0,000385308	3,536989146
ENSG00000159388.5	BTG2	0,000397127	-1,149873187
ENSG00000167165.18	UGT1A6	0,000404859	-1,691359228
ENSG00000081818.3	PCDHB4	0,000457398	2,794929347
ENSG00000113319.12	RASGRF2	0,000533816	1,710009173
ENSG00000165633.12	VSTM4	0,000596581	1,339410459
ENSG00000189190.9	ZNF600	0,000604131	-3,216992944
ENSG00000168004.9	HRASLS5	0,000622107	1,20455123
ENSG00000211448.11	DIO2	0,000622107	-3,323463031
ENSG00000089127.12	OAS1	0,000688351	-2,99949137
ENSG00000186297.11	GABRA5	0,000747108	1,400506461
ENSG00000163032.11	VSNL1	0,000758425	1,284131796
ENSG00000172318.5	B3GALT1	0,000770446	-1,610042792
ENSG00000229544.8	NKX1-2	0,000770446	1,959060458
ENSG00000282278.1	AC058822.1	0,000770446	-2,233200866
ENSG00000205835.8	GMNC	0,000788024	4,856768281
ENSG00000276219.4	ZNF676	0,000788024	-6,06732207
ENSG00000151136.14	BTBD11	0,000912008	-1,572916627
ENSG00000243955.5	GSTA1	0,000935475	-3,654593704
ENSG00000205413.7	SAMD9	0,001042652	2,07450292
ENSG00000137968.16	SLC44A5	0,001131598	-1,958373361
ENSG00000113749.7	HRH2	0,001276119	-3,526231058
ENSG00000205111.8	CDKL4	0,001359424	1,967688492
ENSG00000053747.15	LAMA3	0,001405376	1,521509372
ENSG00000113140.10	SPARC	0,001484351	-2,86464053
ENSG00000102271.13	KLHL4	0,001643973	2,010452644
ENSG00000183850.13	ZNF730	0,001765995	-2,377258953
ENSG00000119938.8	PPP1R3C	0,001853839	1,575247811
ENSG00000112769.18	LAMA4	0,001959756	1,465163464
ENSG00000138061.11	CYP1B1	0,002004429	-2,361041651
ENSG00000111801.15	BTN3A3	0,002042605	1,031999121

ENSG00000149050.9	ZNF214	0,002103324	-2,132499113
ENSG00000133124.11	IRS4	0,002337825	2,078179378
ENSG00000136098.16	NEK3	0,002337825	2,289976578
ENSG00000182985.17	CADM1	0,002351014	1,552910925
ENSG00000065675.14	PRKCQ	0,002390236	1,404662558
ENSG00000185008.17	ROBO2	0,002675836	0,997378516
ENSG00000244509.3	APOBEC3C	0,002806163	-1,13570718
ENSG00000120251.18	GRIA2	0,002963324	-2,382231603
ENSG00000119888.10	EPCAM	0,003005482	1,428593856
ENSG00000180828.2	BHLHE22	0,003005482	1,290302922
ENSG00000204131.9	NHSL2	0,003043956	-1,291865598
ENSG00000184613.10	NELL2	0,003341412	1,387416355
ENSG00000188153.12	COL4A5	0,003439174	-0,984517662
ENSG00000078053.16	AMPH	0,003461591	2,942075355
ENSG00000196335.12	STK31	0,003491305	1,389314512
ENSG00000154065.16	ANKRD29	0,003757987	2,243705688
ENSG00000140285.9	FGF7	0,003791218	1,550436913
ENSG00000127329.15	PTPRB	0,003849477	-1,978158485
ENSG00000176222.8	ZNF404	0,004178641	1,583127749
ENSG00000086205.16	FOLH1	0,00469019	1,374693554
ENSG00000122584.12	NXPH1	0,00469019	-2,334785354
ENSG00000213809.8	KLRK1	0,00469019	1,249593937
ENSG00000134709.10	HOOK1	0,004777414	2,047441004
ENSG00000168016.13	TRANK1	0,00479633	2,458034466
ENSG00000147255.18	IGSF1	0,004978066	-1,090399346
ENSG00000162630.5	B3GALT2	0,004978066	1,623865308
ENSG00000067715.13	SYT1	0,00499675	1,757855655
ENSG00000077092.18	RARB	0,004999942	1,576625887
ENSG00000112837.16	TBX18	0,004999942	-5,288557906
ENSG00000138759.18	FRAS1	0,005381912	1,226231689
ENSG00000197410.12	DCHS2	0,005712886	1,543210134
ENSG00000284227.1	DCHS2	0,005712886	1,543210134
ENSG00000261263.2	AC138907.5	0,006104556	3,660686114
ENSG00000179431.6	FJX1	0,006454915	-1,139359241
ENSG00000117148.7	ACTL8	0,00663589	-2,058487045
ENSG00000068781.21	STON1-GTF2A1L	0,007091077	-1,530340732
ENSG00000166349.9	RAG1	0,007091077	-2,38980673
ENSG00000091490.10	SEL1L3	0,007276661	1,323786096
ENSG00000079257.7	LXN	0,007430446	-1,455886706
ENSG00000213424.8	KRT222	0,007430446	3,92980483
ENSG00000163565.18	IFI16	0,007672389	-1,621112495
ENSG00000074527.11	NTN4	0,007723238	1,102125221
ENSG00000123388.4	HOXC11	0,007843727	2,185322134
ENSG00000165092.12	ALDH1A1	0,007845648	2,631035728
ENSG00000169439.11	SDC2	0,007965431	-1,28550149
ENSG00000244119.1	PDCL3P4	0,008066443	1,389285572
ENSG00000164266.10	SPINK1	0,008167808	-3,989197129

ENSG00000198719.8	DLL1	0,008228801	2,136544537
ENSG00000275555.2	DLL1	0,008228801	2,136544537
ENSG00000105928.13	GSDME	0,008387249	1,06851094
ENSG00000006377.10	DLX6	0,008437771	-1,049533065
ENSG00000179750.15	APOBEC3B	0,008596224	-3,868439489
ENSG00000125820.5	NKX2-2	0,008653705	-1,380674532
ENSG00000169908.11	TM4SF1	0,00866468	1,148711305
ENSG00000143341.11	HMCN1	0,008726876	-3,604061358
ENSG00000186265.9	BTLA	0,009176583	-2,299666805
ENSG00000120451.10	SNX19	0,009775793	0,927451978
ENSG00000112414.14	ADGRG6	0,010450881	2,075211762
ENSG00000203995.9	ZYG11A	0,010591727	1,283051851
ENSG00000176406.22	RIMS2	0,010685455	1,107488186
ENSG00000171956.6	FOXB1	0,010874957	-3,476797352
ENSG00000120907.17	ADRA1A	0,011202236	1,125846664
ENSG00000188112.8	C6orf132	0,012274813	-1,608771646
ENSG00000111432.4	FZD10	0,012640992	-1,070572935
ENSG00000166801.15	FAM111A	0,012660725	1,418852278
ENSG00000196968.10	FUT11	0,013456905	-0,982191362
ENSG00000090530.9	P3H2	0,013542039	1,720769037
ENSG00000163673.7	DCLK3	0,014176021	3,68226767
ENSG00000060140.8	STYK1	0,014330222	1,380507417
ENSG00000155754.14	C2CD6	0,014611145	-4,618905427
ENSG00000133401.15	PDZD2	0,014685056	1,003886711
ENSG00000260062.3	GOLGA2P11	0,014766051	1,325292208
ENSG00000157502.13	MUM1L1	0,015653392	-3,822970846
ENSG00000153246.12	PLA2R1	0,016058359	2,138555486
ENSG00000138670.17	RASGEF1B	0,016157999	-1,210011793
ENSG00000153253.16	SCN3A	0,016157999	2,797488216
ENSG00000145730.20	PAM	0,016165705	1,229168484
ENSG00000204442.3	FAM155A	0,016220404	-1,834370461
ENSG00000225968.7	ELFN1	0,016473905	-5,829227834
ENSG00000135324.5	MRAP2	0,01765477	1,199267338
ENSG00000112276.13	BVES	0,017767415	-1,604779155
ENSG00000176928.5	GCNT4	0,018002208	-1,84712385
ENSG00000231672.6	DIRC3	0,018002208	1,676155751
ENSG00000172020.12	GAP43	0,019231447	-2,290709041
ENSG00000176399.3	DMRTA1	0,019429429	-1,354291702
ENSG00000178568.14	ERBB4	0,019760239	-4,212246387
ENSG00000111846.16	GCNT2	0,019990889	-1,211043557
ENSG00000137462.6	TLR2	0,019990889	1,119006796
ENSG00000227986.1	TRIM60P18	0,021158262	-3,564254497
ENSG00000186462.8	NAP1L2	0,021379115	1,051827322
ENSG00000197013.9	ZNF429	0,021508416	-0,935778361
ENSG00000103241.6	FOXF1	0,02300446	1,798695663
ENSG00000254979.5	AP000781.2	0,02300446	-3,299121634
ENSG00000090376.10	IRAK3	0,023169253	-1,797692153

ENSG00000136158.11	SPRY2	0,023962238	0,818120061
ENSG00000101004.14	NINL	0,024008657	1,097157931
ENSG00000178538.9	CA8	0,024434089	-1,326435603
ENSG00000043355.11	ZIC2	0,024790284	0,996326326
ENSG00000253813.1	COX6B1P4	0,025015792	5,169725673
ENSG00000133134.11	BEX2	0,025306285	-2,931102301
ENSG00000134533.6	RERG	0,026052855	1,759979623
ENSG00000163618.17	CADPS	0,026649045	-1,665255553
ENSG00000134042.12	MRO	0,026809228	1,802202008
ENSG00000111700.12	SLCO1B3	0,028629516	-6,066077898
ENSG00000232196.3	MTRNR2L4	0,028629516	-1,05410956
ENSG00000170989.8	S1PR1	0,029303058	1,043897162
ENSG00000260548.1	AL035425.2	0,029303058	2,531990461
ENSG00000278728.2	CNTNAP2	0,029303058	-1,125227303
ENSG00000159899.14	NPR2	0,02953039	-0,98212061
ENSG00000281020.2	SNTG2	0,031042594	1,822615613
ENSG00000137463.4	MGARP	0,031661036	-1,54912517
ENSG00000166689.15	PLEKHA7	0,033552025	0,885309058
ENSG00000165084.15	C8orf34	0,033929592	-1,494960488
ENSG00000176658.16	MYO1D	0,03394154	0,933863448
ENSG00000115339.13	GALNT3	0,034371352	1,852468666
ENSG00000123119.11	NECAB1	0,034755775	1,519402396
ENSG00000134595.8	SOX3	0,034755775	3,300365161
ENSG00000085563.14	ABCB1	0,034863264	1,306293237
ENSG00000018236.14	CNTN1	0,035831515	1,409626424
ENSG00000142677.3	IL22RA1	0,036272473	2,729362435
ENSG00000270672.1	MTRNR2L6	0,041227426	-1,300472508
ENSG00000186439.12	TRDN	0,041482819	3,382813362
ENSG00000160191.17	PDE9A	0,041799653	1,338753937
ENSG00000006468.13	ETV1	0,042678167	1,22875416
ENSG00000138400.12	MDH1B	0,042710852	1,73060692
ENSG00000185483.11	ROR1	0,042759482	1,916242643
ENSG00000009694.13	TENM1	0,043080922	2,650562943
ENSG00000013619.13	MAMLD1	0,043439517	-1,081258583
ENSG00000109424.3	UCP1	0,044384336	0,847163089
ENSG00000262560.1	AC018512.1	0,045030957	5,606112451
ENSG00000147256.11	ARHGAP36	0,04512278	-2,023253035
ENSG00000169306.9	IL1RAPL1	0,04512278	1,112562134
ENSG00000158089.14	GALNT14	0,046395156	-2,346225578
ENSG00000221887.5	HMSD	0,046395156	1,140797822
ENSG00000197635.9	DPP4	0,048408542	1,273893706
ENSG00000173705.8	SUSD5	0,048415162	-0,795493213
ENSG00000050327.14	ARHGEF5	0,049195089	1,761509183
ENSG00000163492.14	CCDC141	0,049211479	1,124933876

Supplementary Table S2: Significantly altered GO-terms of the transcriptome analysis comparing HAP1 wild-type and HAP1 KDELR1-KO cells, listed with their term-associated deregulated genes and the corresponding fold-change.

**Gene name**	**Fold-change**	**Gene name**	**Fold-change**
**GO-term: Developmental process**			
ACTL8	-2,06	KLKB1	2,41
ACVR1C	3,15	KLRK1	1,25
ADAM9	-9,77	LAMA3	1,52
ADAMTS20	3,53	LAMA4	1,47
ALDH1A2	2,95	LRGUK	1,77
ALX4	-5,30	MAB21L1	1,69
AMBN	-4,13	MAF	1,29
ANGPTL2	-4,76	MAP2	2,09
ANXA1	1,66	MCTP2	2,93
BCHE	2,14	MKX	3,67
BHLHE22	1,29	MLF1	-3,82
BMP7	-9,18	NEK3	2,29
BNC1	3,34	NELL1	-2,03
BST2	-8,24	NEUROG2	2,19
BTG2	-1,15	NKX1-2	1,96
CABYR	1,89	NKX2-2	-1,38
CADM1	1,50	NLGN4X	-7,72
CD24	1,64	NTN4	1,10
CD36	1,91	PAX6	-1,53
CD44	-3,64	PCDH17	1,38
CHMP4C	-3,20	PCDHB4	2,79
CHODL	3,52	PCDHB5	1,48
CHRM3	2,11	PDGFRA	-1,96
CLDN1	1,31	PITX2	1,95
CNTN3	5,46	PLPPR4	2,80
COL11A1	-5,86	PREX2	-2,06
COL5A2	2,78	PRKCQ	1,40
CXCL12	7,63	PROM1	2,45
CXCL14	-1,63	PTGS2	1,87
CYP1B1	-2,36	PTN	7,99
CYR61	-1,52	PTBRB	-1,98
DAZL	-4,05	RAG1	-2,39
DLL1	2,14	RARB	1,58
DLX6	-1,05	RELN	-2,26
DNER	2,46	RIPK4	3,38
EBF2	4,38	ROBO2	1,00
EFNB2	-2,16	RP1	2,18
EHF	-2,82	RSPO3	-3,85
EMP1	-3,80	S1PR3	2,62
EMX2	3,0	SDC2	-1,29
EN1	-2,30	SERPINI1	2,51
ENC1	-2,36	SHISA2	1,36
ENPEP	-2,79	SHROOM3	-2,17
ENPP2	-4,27	SLCO4C1	2,17
EPCAM	1,43	SNTG2	1,82
EPHA5	7,16	SNX19	0,93

ETS1	1,43	SOX1	2,93
EYA1	2,64	SP8	2,12
FAT3	-4,45	SPARC	-2,86
FGF4	-2,28	ST8SIA4	1,92
FGF7	1,55	SYK	2,31
FJX1	-1,14	SYNE1	1,63
FRAS1	1,23	SYT1	1,76
FUT9	-6,16	TBX18	-5,29
GABRA5	1,40	THBS2	-6,44
GJB6	1,32	TIAM1	3,31
GSTA1	-3,65	TIMP3	1,48
GSTM3	1,59	TLE1	1,74
HAND2	1,79	TLL1	2,78
HCLS1	-8,21	TMEFF2	2,09
HES1	-1,63	TNFRSF21	-1,12
HEY1	5,48	TNMD	-1,61
HOOK1	2,05	TRABD2A	-1,30
HOXB7	-6,76	TRO	-3,39
HOXC11	2,16	VCAN	-2,77
HOXC9	-2,55	VGLL2	6,05
HOXD13	1,56	VIM	-5,03
HPGD	1,87	VWC2	3,82
HRH2	-3,53	WIF1	-7,25
IFI16	-1,62		
**GO-term: Cellular developmental process**			
ACTL8	-2,06	IFI16	-1,62
ACVR1C	3,15	KLRK1	1,25
ADAMTS20	3,53	LAMA3	1,52
ADAM9	-9,77	LAMA4	1,47
ALDH1A2	2,95	LRGUK	1,77
ANXA1	1,66	MAF	1,29
BCHE	2,14	MAP2	2,09
BHLHE22	1,29	MLF1	-3,82
BMP7	-9,18	NEK3	2,29
BTG2	-1,15	NELL1	-2,03
CADM1	1,50	NEUROG2	2,19
CABYR	1,89	NKX2-2	-1,38
CD24	1,64	NLGN4X	-7,72
CD36	1,91	NTN4	1,10
CHODL	3,52	PAX6	-1,53
COL11A1	-5,86	PDGFRA	-1,96
COL5A2	2,78	PITX2	1,95
CXCL12	7,63	PLPPR4	2,80
CXCL14	-1,63	PREX2	-2,06
CYR61	-1,52	PRKCQ	1,40
DAZL	-4,05	PROM1	2,45
DLL1	2,14	PTGS2	1,87
DLX6	-1,05	PTN	7,99
DNER	2,46	RAG1	-2,39
EBF2	4,38	RARB	1,58
EFNB2	-2,16	RELN	-2,26
EHF	-2,82	ROBO2	1,00
EMX2	3,0	RP1	2,18

EN1	-2,30	S1PR3	2,26
ENC1	-2,36	SDC2	-1,29
ENPP2	-4,27	SHROOM3	-2,17
EPCAM	1,43	SLCO4C1	2,17
EPHA5	7,16	SNX19	0,93
ETS1	1,43	SOX1	2,93
EYA1	2,64	SPARC	-2,86
FGF4	-2,28	SYK	2,31
GABRA5	1,40	SYNE1	1,63
GSTA1	-3,65	SYT1	1,76
GSTM3	1,59	TIAM1	3,31
HAND2	1,79	TLL1	2,78
HCLS1	-8,21	TNFRSF21	-1,12
HES1	-1,63	TNMD	-1,61
HEY2	5,48	VCAN	-2,77
HOOK1	2,05	VIM	-5,03
HOXB7	-6,76	VWC2	3,82
HRH2	-3,53	WIF1	-7,25
**GO-term: Biological adhesion**			
ADAM9	-9,77	HLA-DQB1	4,35
ADGRE2	-2,31	KLRK1	1,25
AMBN	-4,13	LAMA3	1,52
ANXA1	1,66	LAMA4	1,47
ARHGAP6	1,98	NLGN4X	-7,72
BMP7	-9,18	PCDH17	1,38
BTLA	-2,30	PCDHB4	2,79
CADM1	1,50	PCDHB5	1,48
CD24	1,64	PDGFRA	-1,96
CD36	1,91	PGM5	1,72
CD44	-3,64	PRKCQ	1,40
CLDN1	1,31	PTN	7,99
CNTN3	5,46	RAG1	-2,39
COL4A6	-4,69	RELN	-2,26
CXCL12	7,63	ROBO2	1,00
CYP1B1	-2,36	SERPINI1	2,51
CYR61	-1,52	SMOC2	-3,56
DCHS2	1,54	SYK	2,31
DLL1	2,14	THBS2	-6,44
EFNB2	-2,16	TIAM1	3,31
EPCAM	1,43	TMEM47	3,54
ETS1	1,43	TNFRSF21	-1,12
FAT3	-4,45	TRO	-3,39
HES1	-1,63	VCAN	-2,77
HHLA2	-2,08	VWC2	3,82
**GO-term: Regulation of cell adhesion**			
ADAM9	-9,77	HES1	-1,63
ANXA1	1,66	HHLA2	-2,08
ARHGAP6	1,98	HLA-DQB1	4,35
BMP7	-9,18	KLRK1	1,25
BTLA	-2,30	LAMA3	1,52
CD24	1,64	LAMA4	1,47
CD36	1,91	PRKCQ	1,40

CD44	-3,64	PTN	7,99
CXCL12	7,63	RAG1	-2,39
CYP1B1	-2,36	SERPINI1	2,51
CYR61	-1,52	SMOC2	-3,56
DLL1	2,14	SYK	2,31
EFNB2	-2,16	TNFRSF21	-1,12
EPCAM	1,43	VWC2	3,82
ETS1	1,43		
**GO-term: Multicellular organismal process**			
ACVR1C	3,15	IL20RA	1,64
ADAM9	-9,77	KCNMB2	-1,79
ALB	2,39	KLKB1	2,41
ALDH1A2	2,95	KLRK1	1,25
ALX4	-5,30	LAMA3	1,52
AMBN	-4,13	LAMA4	1,47
ANGPTL2	-4,76	LRGUK	1,77
ANXA1	1,66	LXN	-1,46
BCHE	2,14	LY96	2,83
BHLHE22	1,29	MAB21L1	1,69
BMP7	-9,18	MAF	1,29
BST2	-8,24	MAP2	2,09
BTG2	-1,15	MCTP2	2,93
CABYR	1,89	MKX	3,69
CADM1	1,50	MLF1	-3,82
CD24	1,64	MYOF	-2,69
CD36	1,91	MYOM1	1,83
CD38	2,96	NEK3	2,29
CD44	-3,64	NELL1	-2,03
CHMP4C	-3,20	NEUROG2	2,19
CHODL	3,52	NKX1-2	1,96
CHRM3	2,11	NKX2-2	-1,38
CLDN1	1,31	NLGN4X	-7,72
CNTN3	5,46	NTN4	1,10
COL11A1	-5,86	PAX6	-1,53
COL5A2	2,78	PCDH17	1,38
CSMD1	-1,86	PCDHB4	2,79
CXCL12	7,63	PCDHB5	1,48
CXCL14	-1,63	PDGFRA	-1,96
CYP1B1	-2,36	PITX2	1,95
CYR61	-1,52	PLPPR4	2,80
DAZL	-4,05	PREX2	-2,06
DLL1	2,14	PRKCQ	1,40
DLX6	-1,05	PROM1	2,45
DNER	2,46	PTGS2	1,87
DTNA	-2,49	PTN	7,99
EBF2	4,38	PTPRB	-1,98
EFNB2	-2,16	RAG1	-2,39
EHF	-2,82	RARB	1,58
EMP1	-3,80	RELN	-2,26
EMX2	3,0	ROBO2	1,00
EN1	-2,30	RP1	2,18
ENC1	-2,36	RSPO3	-3,85
ENPEP	-2,79	S1PR3	2,62

ENPP2	-4,27	SDC2	-1,29
EPCAM	1,43	SERPINI1	2,51
EPHA5	7,16	SHISA2	1,36
ETS1	1,43	SHROOM3	-2,05
EYA1	2,64	SLCO4C1	2,17
FAT3	-4,45	SNTG2	1,82
FGF4	-2,28	SNX19	0,93
FGF7	1,55	SOX1	2,93
FJX1	-1,14	SP8	2,12
FRAS1	1,23	SPARC	-2,86
FUT9	-6,16	ST8SIA4	1,92
GABRA5	1,40	SYK	2,31
GJB6	1,32	SYT1	1,76
GLP1R	-3,02	TBX18	-5,29
GSTM3	1,59	THBS2	-6,44
HAND2	1,79	TIAM1	3,31
HCLS1	-8,21	TIMP3	1,48
HES1	-1,63	TLE1	1,74
HEY2	5,48	TLL1	2,78
HHLA2	-2,08	TNFRSF21	-1,12
HMCN1	-3,60	TNMD	-1,61
HOOK1	2,05	TRO	-3,39
HOXB7	-6,76	VCAN	-2,77
HOXC11	2,16	VGLL2	6,05
HOXC9	-2,55	VIM	-5,03
HOXD13	1,56	VWC2	3,82
HPGD	1,87	WIF1	-7,25
HRH2	-3,53		
IFI16	-1,62		
**GO-term: Multicellular organism development**			
ACVR1C	3,15	KLKB1	2,41
ADAM9	-9,77	LAMA3	1,52
ALDH1A2	2,95	LAMA4	1,47
ALX4	-5,30	MAB21L1	1,69
AMBN	-4,13	MAF	1,29
ANGPTL2	-4,76	MAP2	2,09
ANXA1	1,66	MCTP2	2,93
BCHE	2,14	MKX	3,67
BHLHE22	1,29	MLF1	-3,82
BMP7	-9,18	NEK3	2,29
BST2	-8,24	NELL1	-2,03
BTG2	-1,15	NEUROG2	2,19
CADM1	1,50	NKX1-2	1,96
CD24	1,64	NKX2-2	-1,38
CD44	-3,64	NLGN4X	-7,72
CHMP4C	-3,20	NTN4	1,10
CHODL	3,52	PAX6	-1,53
CHRM3	2,11	PCDH17	1,38
CLDN1	1,31	PCDHB4	2,79
CNTN3	5,46	PCDHB5	1,48
COL11A1	-5,86	PDGFRA	-1,96
COL5A2	2,78	PITX2	1,95
CXCL12	7,63	PLPPR4	2,80

CXCL14	-1,63	PREX2	-2,06
CYP1B1	-2,36	PRKCQ	1,40
CYR61	-1,52	PROM1	2,45
DAZL	-4,05	PTGS2	1,87
DLL1	2,14	PTN	7,99
DLX6	-1,05	PTPRB	-1,98
DNER	2,46	RAG1	-2,39
EBF2	4,38	RARB	1,58
EFNB2	-2,16	RELN	-2,26
EHF	-2,82	ROBO2	1,00
EMP1	-3,80	RP1	2,18
EMX2	3,0	RSPO3	-3,85
EN1	-2,30	S1PR3	2,62
ENC1	-2,36	SDC2	-1,29
ENPEP	-2,79	SERPINI1	2,51
ENPP2	-4,27	SHISA2	1,36
EPCAM	1,43	SHROOM3	-2,17
EPHA5	7,16	SLCO4C1	2,17
ETS1	1,43	SNTG2	1,82
EYA1	2,64	SNX19	0,93
FAT3	-4,45	SOX1	2,93
FGF4	-2,28	SP8	2,12
FGF7	1,55	SPARC	-2,86
FJX1	-1,14	ST8SIA4	1,92
FRAS1	1,23	SYK	2,31
FUT9	-6,16	SYT1	1,76
GABRA5	1,40	TBX18	-5,29
GJB6	1,32	THBS2	-6,44
GSTM3	1,59	TIAM1	3,31
HAND2	1,79	TIMP3	1,48
HCLS1	-8,21	TLE1	1,74
HES1	-1,63	TLL1	2,78
HEY2	5,48	TNFRSF21	-1,12
HOOK1	2,05	TNMD	-1,61
HOXB7	-6,76	TRO	-3,39
HOXC11	2,16	VCAN	-2,77
HOXC9	-2,55	VGLL2	6,05
HOXD13	1,56	VIM	-5,03
HPGD	1,87	VWC2	3,82
HRH2	-3,53	WIF1	-7,25
IFI16	-1,62		
**GO-term: Extracellular matrix**			
ADAMTS20	3,53	LAMA4	1,47
ALB	2,39	NTN4	1,10
AMBN	-4,13	PTN	7,99
BMP7	-9,18	RELN	-2,26
COL11A1	-5,86	SMOC2	-3,76
COL4A5	-0,98	SPARC	-2,86
COL4A6	-4,69	SPARCL1	-2,19
COL5A2	2,78	THBS2	-6,44
CYR61	-1,52	TIMP3	1,48
FRAS1	1,23	VCAN	-2,77
HMCN1	-3,60	VWC2	3,82

LAMA3	1,52		
**GO-term: Extracellular matrix component**			
ALB	2,39	LAMA4	1,47
COL11A1	-5,86	NTN4	1,10
COL4A5	-0,98	PTN	7,99
COL4A6	-4,69	SMOC2	-3,76
COL5A2	2,78	SPARC	-2,86
FRAS1	1,23	THBS2	-6,44
HMCN1	-3,60	TIMP3	1,48
LAMA3	1,52	VWC2	3,82
**GO-term: Glycosaminoglycan binding**			
ADGRE2	-2,31	LXN	-1,46
BMP7	-9,18	NELL2	1,39
CD44	-3,64	PTN	7,99
CYR61	-1,52	RSPO3	-3,85
FGF4	-2,28	SMOC2	-3,,76
FGF7	1,55	THBS2	-6,44
VCAN	-2,77		

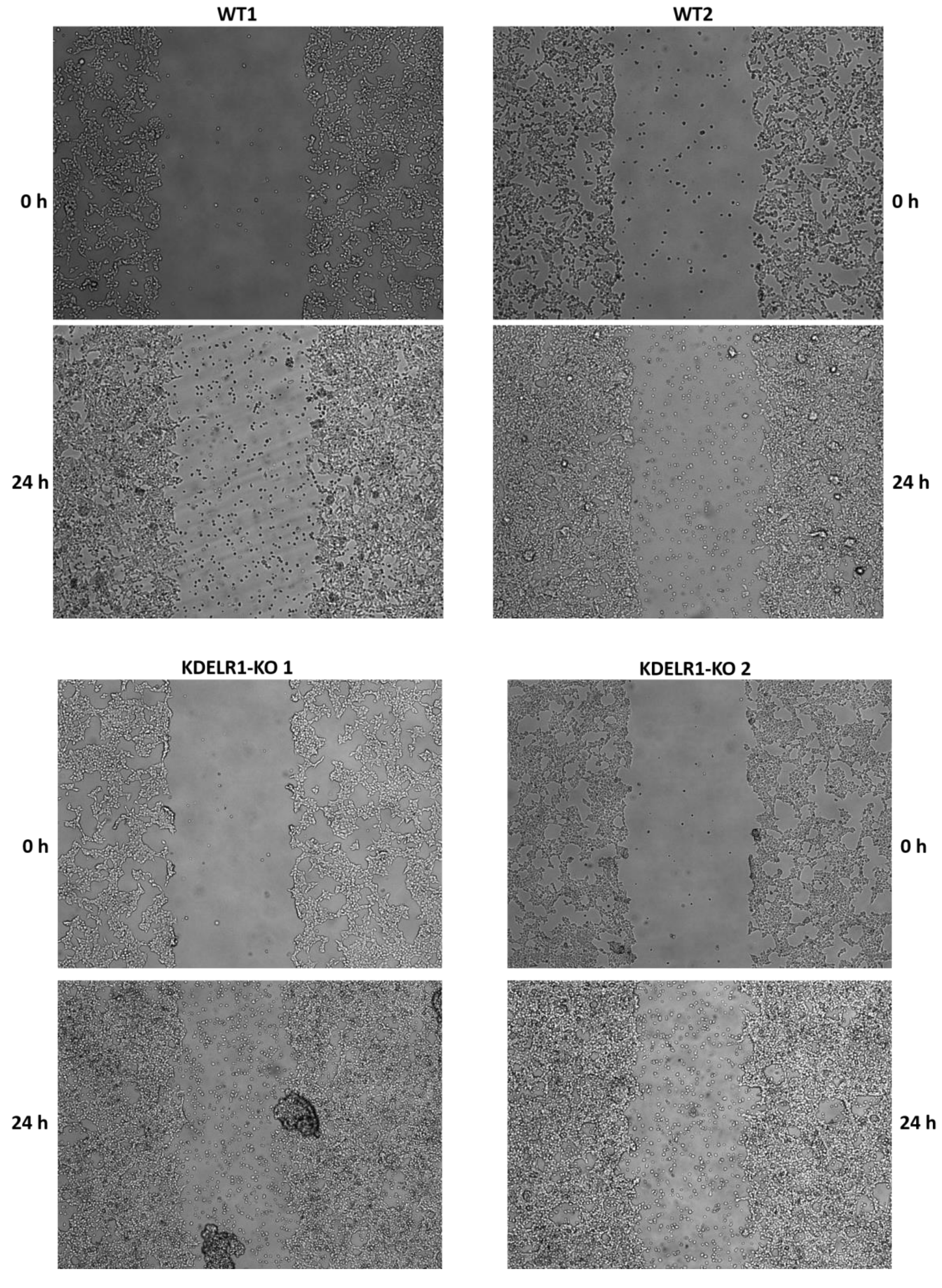

Supplementary Figure S3: Images of HAP1 wild-type and KDELR1-KO cells analysed via *in vivo* scratch assay after 0 and 24 h.
